# Diethyl 3,4-bis(acetoxy­meth­yl)thieno[2,3-*b*]thio­phene-2,5-dicarboxyl­ate

**DOI:** 10.1107/S1600536809036149

**Published:** 2009-09-12

**Authors:** B. Gunasekaran, R. Sureshbabu, A. K. Mohanakrishnan, G. Chakkaravarthi, V. Manivannan

**Affiliations:** a Department of Physics, AMET University, Kanathur, Chennai 603 112, India; b Department of Organic Chemistry, University of Madras, Guindy Campus, Chennai 600 025, India; c Department of Physics, CPCL Polytechnic College, Chennai 600 068, India; d Department of Research and Development, PRIST University, Vallam, Thanjavur 613 403, Tamil Nadu, India

## Abstract

In the title compound, C_18_H_20_O_8_S_2_, the dihedral angle between the two thio­phene rings is 2.33 (7)°. The methyl C atoms of the ester groups are disordered over two positions; the site-occupancy factors of the terminal methyl C atoms are 0.632 (18):0.368 (18) and 0.623 (17):0.377 (17). The mol­ecular structure is stabilized by weak intra­molecular C—H⋯O inter­actions and the crystal structure is stabilized through weak inter­molecular C—H⋯O inter­actions.

## Related literature

For the biological activity of thio­phene derivatives, see: Graff *et al.* (2005 ▶); Hymete *et al.* (2005 ▶); Tapia *et al.* (2003 ▶); Dallemagne *et al.* (2003 ▶). For related structures see: Dufresne & Skene (2008 ▶); Khan *et al.* (2004 ▶). For graph-set notation see: Bernstein *et al.* (1995 ▶).
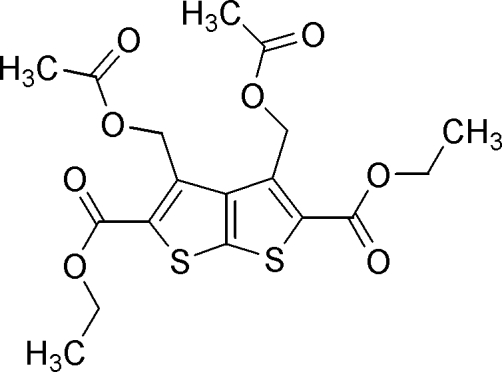


## Experimental

### 

#### Crystal data

C_18_H_20_O_8_S_2_
*M* *_r_* = 428.46
Triclinic, 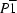

*a* = 9.3214 (5) Å
*b* = 10.2416 (6) Å
*c* = 10.6622 (6) Å
α = 84.952 (3)°
β = 82.814 (4)°
γ = 75.432 (3)°
*V* = 975.72 (9) Å^3^
*Z* = 2
Mo *K*α radiation
μ = 0.32 mm^−1^
*T* = 295 K
0.29 × 0.14 × 0.12 mm

#### Data collection

Bruker Kappa APEXII CCD diffractometer
Absorption correction: multi-scan (*SADABS*; Sheldrick, 1996 ▶) *T*_min_ = 0.914, *T*_max_ = 0.963
25739 measured reflections
6686 independent reflections
4444 reflections with *I* > 2σ(*I*)
*R*_int_ = 0.025

#### Refinement

*R*[*F* ^2^ > 2σ(*F* ^2^)] = 0.057
*wR*(*F* ^2^) = 0.188
*S* = 1.04
6686 reflections
279 parameters
4 restraints
H-atom parameters constrained
Δρ_max_ = 0.82 e Å^−3^
Δρ_min_ = −0.59 e Å^−3^

### 

Data collection: *APEX2* (Bruker, 2004 ▶); cell refinement: *SAINT* (Bruker, 2004 ▶); data reduction: *SAINT*; program(s) used to solve structure: *SHELXS97* (Sheldrick, 2008 ▶); program(s) used to refine structure: *SHELXL97* (Sheldrick, 2008 ▶); molecular graphics: *PLATON* (Spek, 2009 ▶); software used to prepare material for publication: *SHELXL97*.

## Supplementary Material

Crystal structure: contains datablocks global, I. DOI: 10.1107/S1600536809036149/bt5053sup1.cif

Structure factors: contains datablocks I. DOI: 10.1107/S1600536809036149/bt5053Isup2.hkl

Additional supplementary materials:  crystallographic information; 3D view; checkCIF report

## Figures and Tables

**Table 1:** Hydrogen-bond geometry (Å, °)

*D*—H⋯*A*	*D*—H	H⋯*A*	*D*⋯*A*	*D*—H⋯*A*
C10—H10*A*⋯O2	0.97	2.31	2.959 (4)	123
C13—H13*B*⋯O8	0.97	2.32	2.891 (4)	117
C17—H17*C*⋯O8	0.97	2.09	2.563 (5)	108
C10—H10*B*⋯O6^i^	0.97	2.44	3.243 (3)	140
C15—H15*C*⋯O2^ii^	0.96	2.56	3.453 (4)	154

Symmetry codes: (i) 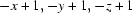; (ii) 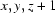.

**Fig. 1.**
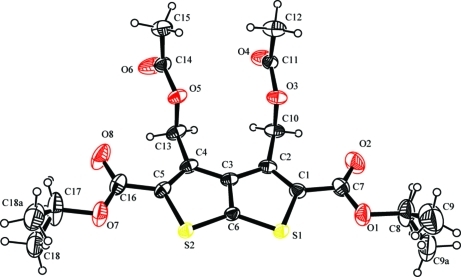
The molecular structure of the title compound, with atom labels and 30% probability displacement ellipsoids for non-H atoms.

**Fig. 2.**
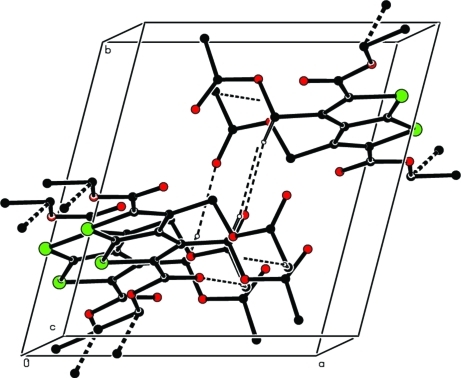
The packing of the title compound, viewed down the C axis. Hydrogen bonds are shown as dashed lines.

## supplementary crystallographic information

### Comment

Thiophene derivatives exhibit anti-inflammatory (Graff *et al.*, 2005; Hymete *et al.*, 2005), anti-protozoal (Tapia *et al.*, 2003) and antitumor (Dallemagne *et al.*, 2003) activities. The geometric parameters of the title compound (Fig. 1) agree with the reported similar structures (Dufresne & Skene, 2008; Khan *et al.*, 2004).

The dihedral angle between the two thiophene rings is 2.33 (7)°. The terminal C atoms of the ester groups are disordered over two positions (the site occupancies of C9 and C9A are 0.37 (2) and 0.63 (2), respectively and C18 and C18A are 0.62 (2) and 0.38 (2), respectively).

The molecular structure is stabilized by weak intramolecular C—H···O interactions and the crystal structure is through weak intermolecular C—H···O interactions. The intramolecular C17—H17C···O8 interaction generate five-membered ring, with graph set motif S(5) and C10—H10A···O2 and C13—H13B···O8 interactions generate six-membered rings, each with graph set motif S(6). The intermolecular C10—H10B···O6 interaction generates an eighteen-membered ring, with graph set motif of *R*_2_^2^(18) (Bernstein *et al.*, 1995).

### Experimental

To a solution of diethyl 3,4-dibromomethylthieno[2,3,-b]thiophene-2,5-dicarboxylate (2 g, 4.25 mmol) in dimethyl formamide (10 ml) was added potassium acetate (1.67 g, 17.0 mmol). The reaction mixture was allowed to stir at room temperature for 5 h under nitrogen atmosphere. The reaction mixture was poured over crushed ice (50 g) containing 1 ml of conc. HCl. The obtained solid was filtered and dried. Recrystallization from methanol afforded the compound.

### Refinement

The site occupancy factors of disordered C atoms were refined as C9 = 0.37 (2), C9A = 0.63 (2), C18 = 0.62 (2) and C18A = 0.38 (2) during anisotropic refinement. The C8—C9, C8—C9A, C17—C18 and C17—C18A bond distances were restrained to be 1.500 (1) Å. H atoms were positioned geometrically and refined using riding model with C—H = 0.97 Å and *U*_iso_(H) = 1.2Ueq(C) for CH_2_, C—H = 0.96 Å and *U*_iso_(H) = 1.5Ueq(C) for CH_3_.

### Crystal data

**Table:**

C_18_H_20_O_8_S_2_	*Z* = 2
*M**_r_* = 428.46	*F*(000) = 448
Triclinic, *P*1	*D*_x_ = 1.458 Mg m^−^^3^
Hall symbol: -P 1	Mo *K*α radiation, λ = 0.71073 Å
*a* = 9.3214 (5) Å	Cell parameters from 5319 reflections
*b* = 10.2416 (6) Å	θ = 2.7–31.0°
*c* = 10.6622 (6) Å	µ = 0.32 mm^−^^1^
α = 84.952 (3)°	*T* = 295 K
β = 82.814 (4)°	Needle, colourless
γ = 75.432 (3)°	0.29 × 0.14 × 0.12 mm
*V* = 975.72 (9) Å^3^	

### Data collection

**Table:**

Bruker Kappa APEX2 CCD diffractometer	6686 independent reflections
Radiation source: fine-focus sealed tube	4444 reflections with *I* > 2σ(*I*)
graphite	*R*_int_ = 0.025
Detector resolution: 0 pixels mm^-1^	θ_max_ = 32.0°, θ_min_ = 1.9°
ω and φ scans	*h* = −13→13
Absorption correction: multi-scan (*SADABS*; Sheldrick, 1996)	*k* = −15→13
*T*_min_ = 0.914, *T*_max_ = 0.963	*l* = −15→15
25739 measured reflections	

### Refinement

**Table:**

Refinement on *F*^2^	Primary atom site location: structure-invariant direct methods
Least-squares matrix: full	Secondary atom site location: difference Fourier map
*R*[*F*^2^ > 2σ(*F*^2^)] = 0.057	Hydrogen site location: inferred from neighbouring sites
*wR*(*F*^2^) = 0.188	H-atom parameters constrained
*S* = 1.04	*w* = 1/[σ^2^(*F*_o_^2^) + (0.0945*P*)^2^ + 0.3857*P*] where *P* = (*F*_o_^2^ + 2*F*_c_^2^)/3
6686 reflections	(Δ/σ)_max_ < 0.001
279 parameters	Δρ_max_ = 0.82 e Å^−^^3^
4 restraints	Δρ_min_ = −0.59 e Å^−^^3^

### Fractional atomic coordinates and isotropic or equivalent isotropic displacement parameters (Å^2^)

**Table:**

	*x*	*y*	*z*	*U*_iso_*/*U*_eq_	Occ. (<1)
S1	0.02951 (5)	0.21624 (6)	0.28483 (5)	0.04706 (15)	
S2	−0.08189 (5)	0.30619 (5)	0.55975 (5)	0.04537 (15)	
O1	0.1876 (2)	0.1299 (2)	0.05271 (17)	0.0747 (5)	
O2	0.4020 (2)	0.1825 (3)	0.0622 (2)	0.0920 (7)	
O3	0.51977 (16)	0.15799 (15)	0.36366 (18)	0.0544 (4)	
O4	0.70351 (19)	0.25297 (19)	0.3848 (2)	0.0719 (5)	
O5	0.40617 (17)	0.27623 (16)	0.65075 (16)	0.0533 (4)	
O6	0.5449 (3)	0.4048 (2)	0.7014 (3)	0.0978 (8)	
O7	−0.1209 (3)	0.3926 (2)	0.80902 (18)	0.0821 (6)	
O8	0.1045 (3)	0.4242 (3)	0.8232 (2)	0.1029 (9)	
C1	0.2123 (2)	0.2247 (2)	0.2352 (2)	0.0483 (5)	
C2	0.2772 (2)	0.2731 (2)	0.3227 (2)	0.0463 (4)	
C3	0.1771 (2)	0.30467 (19)	0.4351 (2)	0.0424 (4)	
C4	0.1817 (2)	0.3531 (2)	0.5561 (2)	0.0469 (5)	
C5	0.0511 (3)	0.3560 (2)	0.6319 (2)	0.0483 (5)	
C6	0.0398 (2)	0.2773 (2)	0.42631 (19)	0.0406 (4)	
C7	0.2789 (3)	0.1791 (3)	0.1093 (2)	0.0592 (6)	
C8	0.2451 (5)	0.0815 (5)	−0.0725 (3)	0.0994 (12)	
H8A	0.3523	0.0700	−0.0854	0.119*	0.368 (18)
H8B	0.2009	0.1469	−0.1369	0.119*	0.368 (18)
H8C	0.2708	0.1540	−0.1285	0.119*	0.632 (18)
H8D	0.3340	0.0087	−0.0667	0.119*	0.632 (18)
C9	0.208 (3)	−0.0509 (13)	−0.0829 (17)	0.148 (7)	0.368 (18)
H9A	0.1181	−0.0543	−0.0286	0.223*	0.368 (18)
H9B	0.2880	−0.1235	−0.0579	0.223*	0.368 (18)
H9C	0.1924	−0.0595	−0.1689	0.223*	0.368 (18)
C9A	0.1272 (9)	0.0290 (14)	−0.1206 (8)	0.112 (4)	0.632 (18)
H9A1	0.1651	−0.0102	−0.2003	0.168*	0.632 (18)
H9A2	0.0413	0.1020	−0.1316	0.168*	0.632 (18)
H9A3	0.0998	−0.0385	−0.0606	0.168*	0.632 (18)
C10	0.4373 (2)	0.2825 (2)	0.3058 (3)	0.0538 (5)	
H10A	0.4724	0.2915	0.2167	0.065*	
H10B	0.4480	0.3596	0.3472	0.065*	
C11	0.6524 (2)	0.1574 (2)	0.3979 (2)	0.0457 (4)	
C12	0.7265 (3)	0.0225 (3)	0.4536 (3)	0.0755 (8)	
H12A	0.7131	−0.0469	0.4044	0.113*	
H12B	0.6830	0.0110	0.5391	0.113*	
H12C	0.8310	0.0164	0.4532	0.113*	
C13	0.3113 (3)	0.3958 (2)	0.5957 (3)	0.0562 (6)	
H13A	0.3653	0.4326	0.5231	0.067*	
H13B	0.2769	0.4644	0.6572	0.067*	
C14	0.5192 (2)	0.2971 (2)	0.7048 (2)	0.0508 (5)	
C15	0.6043 (3)	0.1712 (3)	0.7674 (3)	0.0684 (7)	
H15A	0.7092	0.1640	0.7472	0.103*	
H15B	0.5792	0.0946	0.7381	0.103*	
H15C	0.5796	0.1736	0.8575	0.103*	
C16	0.0166 (4)	0.3957 (3)	0.7634 (2)	0.0638 (6)	
C17	−0.1557 (5)	0.4318 (5)	0.9381 (3)	0.1211 (17)	
H17A	−0.1449	0.5229	0.9429	0.145*	0.368 (18)
H17B	−0.0876	0.3714	0.9916	0.145*	0.368 (18)
H17C	−0.0621	0.4205	0.9736	0.145*	0.632 (18)
H17D	−0.2071	0.5267	0.9393	0.145*	0.632 (18)
C18	−0.3127 (7)	0.4253 (12)	0.9829 (7)	0.097 (3)	0.623 (17)
H18A	−0.3228	0.3352	0.9769	0.146*	0.623 (17)
H18B	−0.3796	0.4871	0.9311	0.146*	0.623 (17)
H18C	−0.3361	0.4495	1.0693	0.146*	0.623 (17)
C18A	−0.238 (2)	0.3432 (15)	1.0223 (10)	0.107 (5)	0.377 (17)
H18D	−0.2882	0.3907	1.0951	0.161*	0.377 (17)
H18E	−0.1680	0.2625	1.0491	0.161*	0.377 (17)
H18F	−0.3090	0.3197	0.9766	0.161*	0.377 (17)

### Atomic displacement parameters (Å^2^)

**Table:**

	*U*^11^	*U*^22^	*U*^33^	*U*^12^	*U*^13^	*U*^23^
S1	0.0368 (2)	0.0638 (3)	0.0455 (3)	−0.0180 (2)	−0.00627 (18)	−0.0101 (2)
S2	0.0411 (2)	0.0553 (3)	0.0445 (3)	−0.0177 (2)	−0.00618 (19)	−0.0085 (2)
O1	0.0760 (12)	0.1018 (15)	0.0518 (10)	−0.0349 (11)	0.0117 (9)	−0.0219 (10)
O2	0.0605 (12)	0.1266 (19)	0.0884 (15)	−0.0303 (12)	0.0247 (11)	−0.0275 (14)
O3	0.0380 (7)	0.0491 (8)	0.0796 (11)	−0.0164 (6)	−0.0142 (7)	0.0062 (7)
O4	0.0470 (9)	0.0678 (11)	0.1098 (16)	−0.0236 (8)	−0.0231 (9)	−0.0044 (10)
O5	0.0529 (8)	0.0509 (8)	0.0645 (10)	−0.0197 (7)	−0.0264 (7)	0.0003 (7)
O6	0.0878 (15)	0.0713 (13)	0.158 (2)	−0.0391 (11)	−0.0728 (16)	0.0083 (14)
O7	0.1042 (16)	0.1005 (15)	0.0523 (10)	−0.0420 (13)	0.0034 (10)	−0.0294 (10)
O8	0.129 (2)	0.140 (2)	0.0676 (13)	−0.0686 (18)	−0.0257 (13)	−0.0312 (14)
C1	0.0373 (9)	0.0561 (12)	0.0520 (11)	−0.0139 (8)	−0.0019 (8)	−0.0022 (9)
C2	0.0342 (8)	0.0440 (10)	0.0619 (13)	−0.0129 (7)	−0.0082 (8)	0.0040 (9)
C3	0.0367 (8)	0.0394 (9)	0.0552 (11)	−0.0144 (7)	−0.0140 (8)	0.0029 (8)
C4	0.0493 (10)	0.0414 (10)	0.0585 (12)	−0.0200 (8)	−0.0232 (9)	0.0021 (8)
C5	0.0560 (11)	0.0487 (11)	0.0474 (11)	−0.0197 (9)	−0.0174 (9)	−0.0040 (8)
C6	0.0351 (8)	0.0462 (10)	0.0440 (10)	−0.0136 (7)	−0.0092 (7)	−0.0034 (8)
C7	0.0542 (12)	0.0617 (14)	0.0585 (14)	−0.0143 (11)	0.0064 (10)	−0.0038 (11)
C8	0.118 (3)	0.129 (3)	0.0556 (17)	−0.046 (3)	0.0230 (18)	−0.0279 (19)
C9	0.175 (18)	0.139 (13)	0.103 (11)	0.008 (13)	0.002 (11)	−0.009 (10)
C9A	0.131 (7)	0.137 (8)	0.078 (5)	−0.048 (6)	0.011 (4)	−0.048 (5)
C10	0.0348 (9)	0.0542 (12)	0.0741 (15)	−0.0175 (8)	−0.0085 (9)	0.0105 (10)
C11	0.0328 (8)	0.0577 (12)	0.0477 (11)	−0.0132 (8)	−0.0030 (7)	−0.0046 (9)
C12	0.0527 (13)	0.0770 (18)	0.091 (2)	−0.0086 (12)	−0.0177 (13)	0.0174 (15)
C13	0.0596 (12)	0.0508 (12)	0.0706 (15)	−0.0270 (10)	−0.0316 (11)	0.0037 (10)
C14	0.0434 (10)	0.0628 (13)	0.0514 (12)	−0.0186 (9)	−0.0118 (8)	−0.0057 (10)
C15	0.0544 (13)	0.0790 (17)	0.0714 (17)	−0.0135 (12)	−0.0198 (12)	0.0090 (13)
C16	0.0896 (19)	0.0617 (14)	0.0500 (13)	−0.0309 (13)	−0.0161 (12)	−0.0082 (11)
C17	0.176 (5)	0.139 (4)	0.064 (2)	−0.071 (3)	0.028 (2)	−0.050 (2)
C18	0.103 (5)	0.136 (8)	0.059 (4)	−0.038 (5)	0.005 (3)	−0.033 (4)
C18A	0.135 (12)	0.115 (10)	0.071 (6)	−0.025 (9)	−0.014 (7)	−0.018 (6)

### Geometric parameters (Å, °)

**Table:**

S1—C6	1.705 (2)	C9—H9A	0.9600
S1—C1	1.741 (2)	C9—H9B	0.9600
S2—C6	1.703 (2)	C9—H9C	0.9600
S2—C5	1.736 (2)	C9A—H9A1	0.9600
O1—C7	1.319 (3)	C9A—H9A2	0.9600
O1—C8	1.455 (3)	C9A—H9A3	0.9600
O2—C7	1.200 (3)	C10—H10A	0.9700
O3—C11	1.331 (2)	C10—H10B	0.9700
O3—C10	1.443 (3)	C11—C12	1.489 (3)
O4—C11	1.183 (3)	C12—H12A	0.9600
O5—C14	1.332 (2)	C12—H12B	0.9600
O5—C13	1.444 (3)	C12—H12C	0.9600
O6—C14	1.182 (3)	C13—H13A	0.9700
O7—C16	1.320 (4)	C13—H13B	0.9700
O7—C17	1.443 (4)	C14—C15	1.485 (3)
O8—C16	1.203 (3)	C15—H15A	0.9600
C1—C2	1.362 (3)	C15—H15B	0.9600
C1—C7	1.471 (3)	C15—H15C	0.9600
C2—C3	1.431 (3)	C17—C18	1.4972 (10)
C2—C10	1.507 (3)	C17—C18A	1.4987 (10)
C3—C6	1.393 (2)	C17—H17A	0.9700
C3—C4	1.431 (3)	C17—H17B	0.9700
C4—C5	1.369 (3)	C17—H17C	0.9700
C4—C13	1.503 (3)	C17—H17D	0.9700
C5—C16	1.469 (3)	C18—H18A	0.9600
C8—C9A	1.4988 (10)	C18—H18B	0.9600
C8—C9	1.4994 (10)	C18—H18C	0.9600
C8—H8A	0.9700	C18A—H18D	0.9600
C8—H8B	0.9700	C18A—H18E	0.9600
C8—H8C	0.9700	C18A—H18F	0.9600
C8—H8D	0.9700		
			
C6—S1—C1	90.24 (10)	C2—C10—H10A	110.7
C6—S2—C5	89.62 (10)	O3—C10—H10B	110.7
C7—O1—C8	115.4 (2)	C2—C10—H10B	110.7
C11—O3—C10	117.10 (16)	H10A—C10—H10B	108.8
C14—O5—C13	115.27 (17)	O4—C11—O3	123.4 (2)
C16—O7—C17	112.2 (3)	O4—C11—C12	125.0 (2)
C2—C1—C7	127.8 (2)	O3—C11—C12	111.5 (2)
C2—C1—S1	113.60 (17)	C11—C12—H12A	109.5
C7—C1—S1	118.60 (17)	C11—C12—H12B	109.5
C1—C2—C3	111.29 (17)	H12A—C12—H12B	109.5
C1—C2—C10	123.9 (2)	C11—C12—H12C	109.5
C3—C2—C10	124.7 (2)	H12A—C12—H12C	109.5
C6—C3—C4	111.15 (19)	H12B—C12—H12C	109.5
C6—C3—C2	111.75 (18)	O5—C13—C4	106.55 (16)
C4—C3—C2	137.07 (18)	O5—C13—H13A	110.4
C5—C4—C3	110.97 (17)	C4—C13—H13A	110.4
C5—C4—C13	124.0 (2)	O5—C13—H13B	110.4
C3—C4—C13	125.0 (2)	C4—C13—H13B	110.4
C4—C5—C16	126.5 (2)	H13A—C13—H13B	108.6
C4—C5—S2	114.14 (16)	O6—C14—O5	122.2 (2)
C16—C5—S2	119.35 (19)	O6—C14—C15	125.9 (2)
C3—C6—S2	114.09 (15)	O5—C14—C15	111.9 (2)
C3—C6—S1	113.12 (15)	C14—C15—H15A	109.5
S2—C6—S1	132.76 (11)	C14—C15—H15B	109.5
O2—C7—O1	122.8 (3)	H15A—C15—H15B	109.5
O2—C7—C1	125.5 (3)	C14—C15—H15C	109.5
O1—C7—C1	111.7 (2)	H15A—C15—H15C	109.5
O1—C8—C9A	107.7 (3)	H15B—C15—H15C	109.5
O1—C8—C9	108.9 (7)	O8—C16—O7	124.1 (3)
O1—C8—H8A	109.9	O8—C16—C5	124.0 (3)
C9A—C8—H8A	139.2	O7—C16—C5	111.9 (2)
C9—C8—H8A	109.9	O7—C17—C18	108.9 (3)
O1—C8—H8B	109.9	O7—C17—C18A	113.7 (5)
C9A—C8—H8B	72.5	O7—C17—H17A	109.9
C9—C8—H8B	109.9	C18—C17—H17A	109.9
H8A—C8—H8B	108.3	C18A—C17—H17A	133.7
O1—C8—H8C	110.2	O7—C17—H17B	109.9
C9A—C8—H8C	111.5	C18—C17—H17B	109.9
C9—C8—H8C	138.2	C18A—C17—H17B	70.5
H8A—C8—H8C	69.4	H17A—C17—H17B	108.3
O1—C8—H8D	109.8	O7—C17—H17C	107.6
C9A—C8—H8D	109.2	C18—C17—H17C	138.3
C9—C8—H8D	70.6	C18A—C17—H17C	105.0
H8B—C8—H8D	137.3	H17A—C17—H17C	75.1
H8C—C8—H8D	108.5	O7—C17—H17D	109.6
C8—C9—H9A	109.5	C18—C17—H17D	78.0
H8D—C9—H9A	131.3	C18A—C17—H17D	112.7
C8—C9—H9B	109.5	H17B—C17—H17D	134.0
H8D—C9—H9B	72.5	H17C—C17—H17D	107.8
C8—C9—H9C	109.5	C17—C18—H18A	109.5
H8D—C9—H9C	115.5	C17—C18—H18B	109.5
C8—C9A—H9A1	109.5	C17—C18—H18C	109.5
C8—C9A—H9A2	109.5	C17—C18A—H18D	109.5
H9A1—C9A—H9A2	109.5	C17—C18A—H18E	109.5
C8—C9A—H9A3	109.5	H18D—C18A—H18E	109.5
H9A1—C9A—H9A3	109.5	C17—C18A—H18F	109.5
H9A2—C9A—H9A3	109.5	H18D—C18A—H18F	109.5
O3—C10—C2	105.38 (16)	H18E—C18A—H18F	109.5
O3—C10—H10A	110.7		
			
C6—S1—C1—C2	−0.51 (18)	C1—S1—C6—S2	−176.94 (17)
C6—S1—C1—C7	178.72 (19)	C8—O1—C7—O2	−1.0 (5)
C7—C1—C2—C3	−178.9 (2)	C8—O1—C7—C1	−179.6 (3)
S1—C1—C2—C3	0.2 (2)	C2—C1—C7—O2	−2.8 (4)
C7—C1—C2—C10	−2.9 (4)	S1—C1—C7—O2	178.1 (2)
S1—C1—C2—C10	176.25 (16)	C2—C1—C7—O1	175.8 (2)
C1—C2—C3—C6	0.2 (3)	S1—C1—C7—O1	−3.3 (3)
C10—C2—C3—C6	−175.73 (18)	C7—O1—C8—C9A	179.6 (6)
C1—C2—C3—C4	177.8 (2)	C7—O1—C8—C9	136.4 (11)
C10—C2—C3—C4	1.9 (4)	C11—O3—C10—C2	−159.1 (2)
C6—C3—C4—C5	1.7 (2)	C1—C2—C10—O3	−92.8 (3)
C2—C3—C4—C5	−175.9 (2)	C3—C2—C10—O3	82.6 (3)
C6—C3—C4—C13	−178.15 (18)	C10—O3—C11—O4	1.0 (3)
C2—C3—C4—C13	4.3 (4)	C10—O3—C11—C12	−178.8 (2)
C3—C4—C5—C16	178.0 (2)	C14—O5—C13—C4	−172.7 (2)
C13—C4—C5—C16	−2.2 (4)	C5—C4—C13—O5	91.0 (3)
C3—C4—C5—S2	−1.9 (2)	C3—C4—C13—O5	−89.2 (3)
C13—C4—C5—S2	177.97 (16)	C13—O5—C14—O6	−4.2 (4)
C6—S2—C5—C4	1.19 (17)	C13—O5—C14—C15	175.7 (2)
C6—S2—C5—C16	−178.7 (2)	C17—O7—C16—O8	2.0 (5)
C4—C3—C6—S2	−0.8 (2)	C17—O7—C16—C5	−179.9 (3)
C2—C3—C6—S2	177.42 (14)	C4—C5—C16—O8	−4.2 (4)
C4—C3—C6—S1	−178.87 (14)	S2—C5—C16—O8	175.6 (2)
C2—C3—C6—S1	−0.6 (2)	C4—C5—C16—O7	177.6 (2)
C5—S2—C6—C3	−0.18 (16)	S2—C5—C16—O7	−2.5 (3)
C5—S2—C6—S1	177.39 (17)	C16—O7—C17—C18	−179.8 (6)
C1—S1—C6—C3	0.64 (16)	C16—O7—C17—C18A	−136.2 (10)

### Hydrogen-bond geometry (Å, °)

**Table:**

*D*—H···*A*	*D*—H	H···*A*	*D*···*A*	*D*—H···*A*
C10—H10A···O2	0.97	2.31	2.959 (4)	123
C13—H13B···O8	0.97	2.32	2.891 (4)	117
C17—H17C···O8	0.97	2.09	2.563 (5)	108
C10—H10B···O6^i^	0.97	2.44	3.243 (3)	140
C15—H15C···O2^ii^	0.96	2.56	3.453 (4)	154

Symmetry codes: (i) −*x*+1, −*y*+1, −*z*+1; (ii) *x*, *y*, *z*+1.
